# Anti-malarial activities of *Andrographis paniculata *and *Hedyotis corymbosa *extracts and their combination with curcumin

**DOI:** 10.1186/1475-2875-8-26

**Published:** 2009-02-12

**Authors:** Kirti Mishra, Aditya P Dash, Bijay K Swain, Nrisingha Dey

**Affiliations:** 1 Institute of Life Sciences, Nalco Square, Chandrasekhar Pur, Bhubaneswar – 751 023, Orissa, India; 2 National Institute of Malaria Research, 22 Shamnath Marg, New Delhi – 110 054, India; 3 Silviculture Division, Department of Forests, Govt of Orissa, Ghatikia, Khandagiri, Bhubaneswar, Orissa, India

## Abstract

**Background:**

Herbal extracts of *Andrographis paniculata *(AP) and *Hedyotis corymbosa *(HC) are known as hepato-protective and fever-reducing drugs since ancient time and they have been used regularly by the people in the south Asian sub-continent. Methanolic extracts of these two plants were tested in vitro on choloroquine sensitive (MRC-pf-20) and resistant (MRC-pf-303) strains of *Plasmodium falciparum *for their anti-malarial activity.

**Methods:**

Growth inhibition was determined using different concentrations of these plant extracts on synchronized *P. falciparum *cultures at the ring stage. The interactions between these two plant extracts and individually with curcumin were studied in vitro. The performance of these two herbal extracts in isolation and combination were further evaluated in vivo on Balb/c mice infected with *Plasmodium berghei *ANKA and their efficacy was compared with that of curcumin. The in vivo toxicity of the plant derived compounds as well as their parasite stage-specificity was studied.

**Results:**

The 50% inhibitory concentration (IC_50_) of AP (7.2 μg/ml) was found better than HC (10.8 μg/ml). Combination of these two herbal drugs showed substantial enhancement in their anti-malarial activity. Combinatorial effect of each of these with curcumin also revealed anti-malarial effect. Additive interaction between the plant extracts (AP + HC) and their individual synergism with curcumin (AP+CUR, HC+CUR) were evident from this study. Increased in vivo potency was also observed with the combination of plant extracts over the individual extracts and curcumin. Both the plant extracts were found to inhibit the ring stage of the parasite and did not show any in vivo toxicity, whether used in isolation or in combination.

**Conclusion:**

Both these two plant extracts in combination with curcumin could be an effective, alternative source of herbal anti-malarial drugs.

## Background

The effective anti-malarial activity of the two plant-based drugs, quinine and artemisinin, has generated much interest to explore other plant resources for their possible anti-malarial efficacy. Malaria is still an ever-continuing epidemic that claims thousands of lives in the Indian sub-continent each year and the majority of the malarial deaths are due to *Plasmodium falciparum*. During last decade, several fundamental researches have been conducted to explore anti-malarial activity of many plants, including *Citrus cinensis*, *Carcia papaya*, *Swertia chirata *[1], *Bidens pilosa *[2], *Piper sarmentosum*, *Tinospora corpa *[3] and many others [4]. Curcumin, a natural polyphenolic compound (isolated from root of *Curcuma longa*, turmeric) apart from its diverse pharmacological properties including anticarcinogenic [5], is also known to have anti-protozoan activity [6]. Further, its efficacy has been evaluated and advocated in a combination therapy with artemisinin (derived from plant *Artemisia annua*) to control malaria [7]. Combination drug regimens have become the practice of choice because of their increased therapeutic efficacy over monotherapy and the other benefits include decreased cytotoxicity, delay or prevention of the development of drug resistance [8].

In the present study, *Andrographis paniculata *(Acanthaceae) and *Hedyotis corymbosa *(Rubiaceae) were selected for evaluation of their anti-malarial activities, as their traditional uses as anti-pyretic drugs are common among different tribal population in south-east India. The ethno-pharmacological properties of *A. paniculata *are well documented and several in vitro and in vivo studies describe its anti-cold, anti-hepatotoxic, anti-urothelial and anti-hepato-toxic properties [9-11]. Some studies have also reported its anti-malarial properties [12-15]. In contrast, other than a single study [16] that documents the hepato-protective activity of *H. corymbosa*, there is no report of anti-malarial activity.

This report demonstrates the anti-malarial properties of these two herbal extracts, individually and in combination on *Plasmodium falciparum *in vitro and on *Plasmodium berghei *in vivo; and compares their activity in combination with curcumin. The stage-specific activity of these extracts and their in vivo toxicity has also been investigated. The anti-malarial activities exerted by these two plant extracts were found comparable with that of curcumin.

## Methods

### Plant materials and chemicals

**Figure 1:**
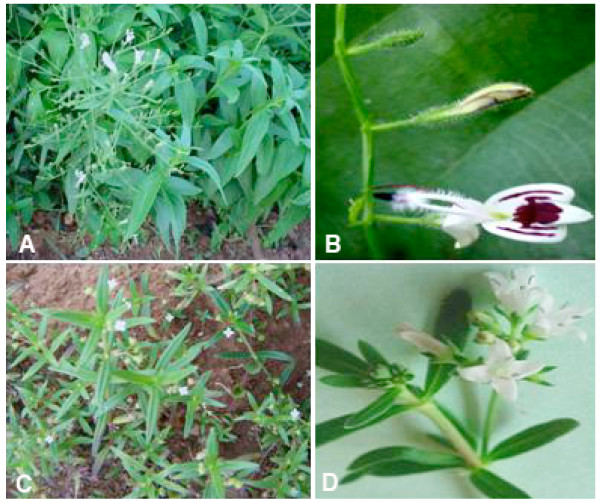
**A flowering plant of *Andrographis paniculata *(A), Flower of *Andrographis paniculata *(B)**. A flowering plant of *Hedyotis corymbosa *(C), A twig of *Hedyotis corymbosa *showing the arrangement of flowers. *Andrographis paniculata *is an herb, usually 30–90 cm in height and commonly found in plains all over India, China and other subtropical countries in South East Asia. *Hedyotis corymbosa *is a small herb, 5–30 cm long, distributed through out India and other subtropical countries. Both of these plants can grow between 25 to 40°C temperatures and at any soil texture.

Two plants, *A. paniculata *and *H. corymbosa *having the taxonomic serial numbers TSN 184881 and TSN 514495, respectively, as per the Integrated Taxonomic Information System (ITIS) were collected from the silviculture nursery of Department of Forests, Government of Orissa, India (Figure 1A–D). The saplings were raised from the pure seeds obtained from the Government nursery and were grown in Green House at 25°C under 16/8 hrs of day night cycle. Pure curcumin (Himedia, Cat # RM 1449) and RPMI 1640 medium (Himedia, Cat # AT 060-1L) were purchased commercially.

### Preparation of plant extract

The aerial parts of the plants were crushed in liquid nitrogen to fine powder. An aliquot of 5.0 gram of pulverized material from each plant was stirred with 98% methanol (50 ml) at 4°C over night and filter-sterilized using cellulose acetate membranes (0.45 μm; Millipore Corporation, Bedford, MA). Individual plant extracts were evaporated to dryness at 37°C by the help of a Speed Vac (Model 7811001, Labconco, USA) and the residue was stored in aliquots at 4°C until tested. Usually, a 0.4% yield of extract was obtained invariably from each of the plants.

### Parasite strain and in vitro culture

Two strains of *P. falciparum*, one sensitive to chloroquine (MRC-pf-20) and the other resistant (MRC-pf-303) were obtained from the Malaria Parasite bank, maintained by the National Institute of Malaria Research, New Delhi, India. The cultures have been maintained in the laboratory using the candle jar method [17] in human red blood cells (blood type O^+^) at a 5% haematocrit in RPMI 1640 medium with 25 mM HEPES, 0.2% sodium bicarbonate and 15% human AB^+ ^serum. The ANKA strain of *P. berghei *was procured as a generous gift from the Tata Institute of Fundamental Research, Mumbai, India.

### Determination of dose response of *A. paniculata *and *H. corymbosa *extracts on in vitro grown *P. falciparum*

The parasite cultures, prior to experimentation, were synchronized by treatment with 5% D-sorbitol [18]. Synchronized cultures containing ring-staged parasites were suspended in equal volume of human serum. Stock solutions were prepared separately from 10 mg of dried herbal extract of these two plants and from the commercial grade curcumin, by dissolving them in minimum volume (10 μl) of dimethyl sulfoxide (DMSO) and finally diluting with serum free medium to a concentration of 1 mg/ml. Serial double dilutions of each set of plant extracts were made in triplicate in 96 well microtitre plates (Axygen, Germany) with concentration ranging from 1.8–500 μg/ml against a control containing the incomplete medium with same concentration of DMSO. In each well 100 μl of the diluted extract, 10 μl parasitized blood (4 – 5% rings) in 100 μl incomplete medium and 5% haematocrit were added. Four wells of the last row were set as general controls to check the normal growth of the parasite. Schizont maturation time was calculated from the growth of the parasites cultured in general control wells. Accordingly thin smears were drawn (approximately 24–28 hrs of incubation) from each of the experimental and control wells on properly labeled slides. The blood smears were air dried and fixed in methanol. Dried slides were JSB-stained [19] and observed in 100 × with oil immersion under microscope (DM LS2; Leica) for the study of parasitaemia, particularly the inhibition of schizont maturation. Numbers of schizonts were counted per 200 asexual stage parasites. The values were compared between test and control wells. The percentage of inhibition was calculated as (1-Number of schizonts in test well/Number of schizonts per control well) × 100. The 50% inhibitory concentration (IC_50_) of these plant extracts were estimated from the graph drawn on the inhibition (%) data (Table 1).

**Table 1:** Percent (%) inhibition of schizonts by extracts of *Andrographis paniculata *(AP) and *Hedyotis corymbosa *(HC) in choloroquine sensitive (MRC-pf-20) and resistant strains (MRC-pf-303) of *Plasmodium falciparum*.

Plant extracts	Concentration of drug (μg/ml)	Chloroquine sensitive (MRC-pf-20)	Chloroquine resistant (MRC-pf-303)
*Andrographis paniculata*	1.8	15.3 ± 1.49	11.6 ± 0.6
	
	3.6	33.6 ± 2.1	28.13 ± 0.6
	
	7.2	52.6 ± 1.01	50.7 ± 1.3
	
	15.0	70.5 ± 1.9	66.1 ± 0.8
	
	30.0	85.3 ± 1.5	81.6 ± 1.8
	
	62.5	96. 8 ± 0.7	94.8 ± 0.8
	
	125	100	100
	
	250	100	100
	
	500	100	100

*Hedyotis corymbosa*	1.8	8.3 ± 1.2	6.7 ± 0.7
	
	3.6	20.2 ± 0.8	17.5 ± 1.4
	
	7.2	39.3 ± 0.9	33.1 ± 0.6
	
	15.0	61.7 ± 1.1	59.8 ± 2.0
	
	30.0	82.4 ± 1.9	77.3 ± 1.3
	
	62.5	91.8 ± 0.6	88.7 ± 0.7
	
	125	97.5 ± 0.9	96.9 ± 0.05
	
	250	100	100
	
	500	100	100

### In vitro interaction of plant extracts and their combination with curcumin

Drug interaction studies were performed as described [20] on chloroquine resistant isolates of *P. falciparum *(MRC-pf-303). The IC_50 _value of pure curcumin (CUR) was calculated as 6.6 μg/ml [7]. For interaction studies, putative drug solutions of *A. paniculata *(AP), *H. corymbosa *(HC) and curcumin (CUR) were diluted with serum-free culture medium to initial concentrations of 80 times the predetermined IC_50 _(Table 1). These solutions were combined in the ratios of 1:5, 1:2, 2:1 and 5:1 for each interacting trial (AP+HC, AP+CUR and HC+CUR). Similarly combinations of three putative drugs (AP+HC+CUR) were prepared in three ratios (3:2:1, 2:1:3, 1:3:2). The solutions were then introduced in duplicate into 96-well titer plates with serial double dilutions from their respective stock to ensure different concentrations of single and combined drug solutions in each well along with parasites at their ring stage. The plates were incubated at 37°C in the candle jar. Finally, IC_50 _values of individual putative drugs and their combinations were determined. For data interpretation, IC_50 _values of these drugs in combination were expressed as fractions of the IC_50 _of the extracts alone normalized to 1. Isobolograms were constructed by plotting the IC_50 _of one extract against the IC_50 _of the other for each of the four combinations. To express level of interactions numerically, results were represented as the sum of the fractional inhibitory concentrations (sum FIC). FIC = IC_50 _of extract A in mixture/IC_50 _of extract A alone + IC_50 _of extract B in mixture/IC_50 _of extract B alone. Sum FIC values were considered to be the indication of the type of interaction between putative drugs and represented as synergy for the value < 0.5, 0.5 to 1 as addition; 1 to 2 as indifferent interaction and > 2 antagonisms.

### In vivo toxicity assay

Mice (Balb/c, 25 g ± 4 g) were used and randomly distributed into five groups (n = 4/group) for this study. They were kept in filter top cages and housed in environmentally controlled rooms (temperature, 22 ± 0.8°C; relative humidity 30 to 50%) with a 12 h day and night light cycle. Food and water were provided *ad libitum*. Three groups of mice were given intraperitoneal injections (50 mg/kg of body weight) of the drugs AP, HC and (AP + HC) respectively and a group of mice was given DMSO (0.01%) as control experiment, while the other control group was kept without any treatment. Mice were observed frequently at 8–10 h intervals for any discomfort until 72 h post-injection. At the end of three days (72 h), the body weight was recorded. Necropsy was performed and the livers and kidneys were removed for histopathological analysis. For blood analysis, animals were bled through heart puncture and the samples were analysed for Serum Glutamic Pyruvic Transaminase (SGPT) and Serum Glutamic Oxaloacetic Transaminase (SGOT) enzymes by the method described earlier [21]. Hematological analyses were performed from the whole blood by fully auto blood counter (RMS-Hestia 60, Chandigarh, India).

### Stage specific inhibitory effect of herbal extracts on chloroquine resistant strains of *P. falciparum*

The stage-specific inhibitory effect of herbal extracts was studied by following a published protocol [22]. Briefly, ring-infected RBCs (0 to 5 h old), trophozoite-infected RBCs (20 to 25 h old), and schizont-infected RBCs (37 to 42 h old) were incubated in presence of both the plant extracts at their IC_90 _concentrations (60 μg/ml) for 5–6 hour at 37°C. Cells were washed with incomplete medium to remove the drugs after incubation and were then further maintained in regular culture condition. Untreated synchronous controls were processed in the same way as the treated cultures. After 24 hours, blood smears were prepared and JSB-stained. Parasitaemia was established on 2,000 cells by counting number of new rings that formed during the second erythrocytic cycle for both the plant extracts.

### In vivo study on anti-malarial activities of the plant extracts

The in vivo anti-malarial activity of *A. paniculata*, *H. corymbosa *and their combinations were evaluated and compared with that of curcumin by a series of tests. Mice, (Balb/c, 28 g ± 4 g) were randomly distributed into five groups, each group comprising of four animals. The blood of a donor mouse with 30% parasites at old trophozoite stage was collected in a heparinized syringe. The parasitized blood was diluted in physiological saline solution (0.9% NaCl) to a density of 5 × 10^7 ^RBCs/ml.

Mice of all five groups were inoculated intra-peritoneally with 0.2 ml of the parasite suspension (1 × 10^7 ^parasites) of *P. berghei *ANKA on day 1. The test compounds (AP, HC, AP+ HC and CUR) were dissolved in DMSO along with serum free medium to make desired concentrations (7 mg/kg of body weight) and were injected intra-peritoneally to four individual groups of animals designated as AP, HC, AP +HC and CUR, respectively. Drug treatments were initiated 24 h prior to the parasite challenge (day 0). The control experiment, without any drug treatment, was performed simultaneously. Drug treatments were continued on a daily basis until the death of mice noted among the control group. Starting from day-4 of post-infection, on every alternate day, the level of parasitaemia in each group of mice was determined from tail-blood. The animal experiments complied with all relevant guidelines and institutional policies on animal ethics.

## Results

### Growth inhibition assay of *P. falciparum *using *A. paniculata *and *H. corymbosa *extracts

The plant extracts of AP (*A. paniculata*) and HC (*H. corymbosa*) manifested significant activities against both the resistant and sensitive strains of *P. falciparum*. The percentage of growth inhibition was determined using different concentrations of plant extracts (Table 1). The results were compared to that of the parasites grown in control wells (without treatment of herbal extracts). Maximum growth inhibition (97% in chloroquine-sensitive and 95% in chloroquine-resistant strains) was obtained with AP at a concentration of 62.5 μg/ml. The concentration above this (125, 250 and 500 μg/ml) exhibited total growth inhibition of the parasites at ring stage for both the strains. Whereas HC showed its maximum inhibitory effect (97%) at a concentration of 125 μg/ml in both chloroquine-sensitive and -resistant strains (Table 1). Higher concentrations (250 and 500 μg/ml) of HC caused total inhibition of the parasite at ring stage. The IC_50 _values of these plants were calculated and found to be7.2 μg/ml and 10.8 μg/ml for AP and HC, respectively.

### In vitro drug interactions

**Figure 2:**
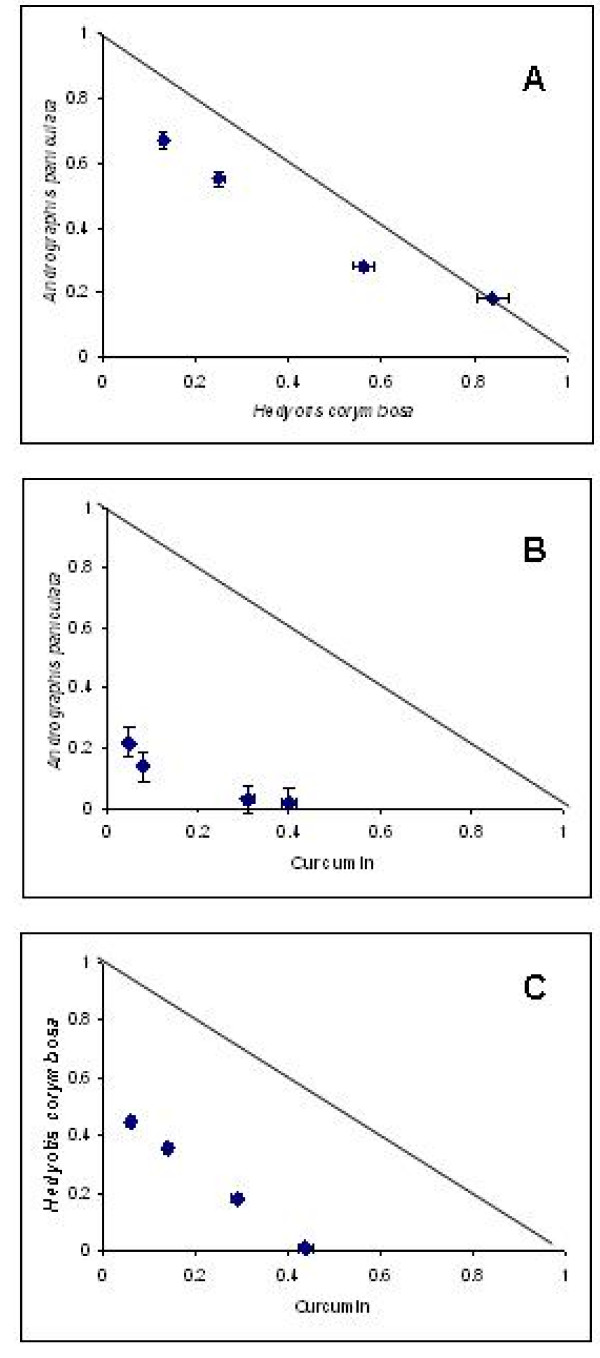
**In vitro interaction of plant extracts studied in choloroquine resistant strains (MRC-pf-303) of *P. falciparum***. Representative isobolograms of the interaction of *Andrographis paniculata *with *Hedyotis Corymbosa *(A), *Andrographis paniculata *with curcumin (B), *Hedyotis corymbosa *with curcumin (C).

As both chloroquine-sensitive (MRC-pf-20) and -resistant (MRC-pf-303) stains of *P. falciparum *exhibited similar response to extracts of both plants, the drug interactions studies were kept limited to the resistant (MRC-pf-303) strain only. AP, HC and curcumin (CUR), in dilutions were assayed individually (as described in Materials and Method section) and IC_50 _values were 9, 13.5 and 8.5 μg/ml, respectively. In vitro interaction of the combined AP+HC and individually combined with curcumin (AP+CUR, HC+CUR) are presented in Figure 2. The IC_50 _values of the individual drugs in four different ratios were significantly lower than the IC_50 _values of the individual drugs resulting in a concave curve in isobologram. The sum FIC values of these combinations representing the numeric value for the type of interaction are represented in Table 2. The combination of AP and HC (Figure 2A) indicate additive interaction, while the combinations, AP+CUR (Figure 2B) and HC+CUR (Figure 2C) were found to be synergistic. Indifferent interactions were found in the tri-combination (AP+HC+CUR) when tested in ratio 2:1:3, while in other two ratios (3:2:1, 1:3:2) were found to be additively interactive.

**Table 2:** Sum FIC_50 _of extracts of *Andrographis paniculata *(AP), *Hedyotis corymbosa *(HC) and curcumin in different combinations

Combinations	Ratios			
	
	1:5	1:2	2:1	5:1
AP+HC	0.8 ± 0.06	0.8 ± 0.04	0.84 ± 0.04	1.02 ± 0.1

AP+CUR	0.27 ± 0.03	0.22 ± 0.04	0.44 ± 0.03	0.34 ± 0.01

HC+CUR	0.45 ± 0.05	0.47 ± 0.04	0.5 ± 0.04	0.51 ± 0.04

### Determination of stage-specific effectiveness of the test plant extracts

The stage-specific effectiveness of AP and HC were determined by applying these putative drugs individually at their IC_90 _concentrations in three different developmental stages, namely, ring-, trophozoite- and schizont-stage of *P. falciparum*, with respective controls. Positive inhibitory effects with both the extracts were found only with ring-stage parasites. The other treatment groups (trophozoites and schizonts) exhibited normal development as the controls. The growth inhibition of the parasites at ring stage was found to be permanent, as the rings underwent lyses and lost the cellular architecture with extension of incubation period from 24 h to 48 h.

### In vivo toxicity study

Follow-up evaluations of herbal drugs both in isolation (AP, HC) and combination (AP+HC) to test the in vivo toxicity in mice showed no obvious toxic side effects and treated mice were found healthy and normal with no record of weight loss, hair loss, allergy or any other symptoms of discomfort. The hematological parameters between the experimental and control sets were the same without any major changes. Sections of liver and kidney did not show significant changes on administration of plant extracts. Liver sections from the control mice as well as from the treated mice that were administered AP, HC, AP + HC showed a normal pattern of hepatic and biliary parenchyma. All the blood parameters remained within the normal range. The levels of SGOT and SGPT in both control as well as in drug treated mice were found almost the same (SGOT, 62.8 ± 5.3; SGPT, 78.3 ± 7.5 U/ml).

### In vivo study on anti-malarial activities of herbal extracts

**Figure 3:**
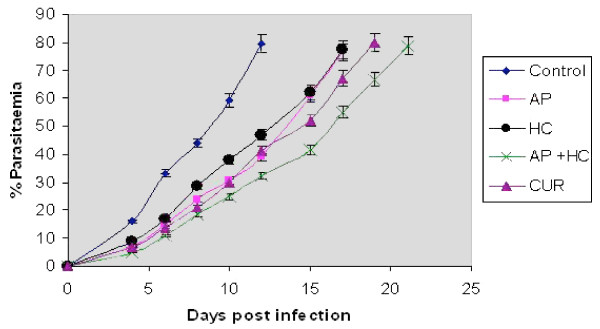
**In vivo efficacy of the extracts of *Andrographis paniculata *(AP), *Hedyotis corymbosa *(HC) in isolation and in combination (AP+HC) compared with curcumin (CUR) in *P. berghei *ANKA infected mice**.

The efficacy of plant extracts (AP, HC and combination of AP+HC) studied in vivo in *P. berghei*-infected Balb/c mice is shown in Figure 3. Their performances were compared with that of curcumin. The drugs were injected daily to experimental groups and the parasitaemia from both experimental and control group was checked on alternate days. The control mice died on or before 13^th ^day and at this point administration of drugs to the experimental groups were discontinued. The average % of parasitaemia in the control group was found 79.5 ± 2.8% on 12^th ^day studied against the experimental groups treated with AP, HC, Curcumin and AP + HC, which exhibited 39%, 47%, 41% and 32% of parasitaemia, respectively. On an average, the day of demise of each treatment group, AP, HC, Curcumin and AP + HC were recorded as 18^th^, 17^th^, 19^th ^and 22^nd ^day of experiments, which was due to high parasitaemia (80 ± 3%), observed ubiquitously (Figure 3).

## Discussion

The present study assessed the anti-malarial properties of two Indian traditional plants, *A. paniculata *and *H. corymbosa*, individually and in combinations and compared their activity with curcumin.

Traditional uses of *A. paniculata *extracts in the Indian medicine particularly in the treatment of various liver disorders are common [4,23-25]. Earlier studies have also demonstrated the antipyretic [26] and anti-diarrheal [27] properties of this plant. The role of Andrographolides, one of its major phytoconstituents has been reported to have inhibitory effect on the rat and human hepatic cytochrome P450 [28] and the alpha-glucosidase and alpha-amylase enzymes that cause type 2 diabetes [29]. Few reports are also available on the anti-malarial activity of the plant *A. paniculata *[3,4,12-15]. However, exploitation of drug potentials of *H. corymbosa *is limited, except one, which document the hepato-protective activity of this plant [16]. This lacuna prompted us to explore the possibility.

Both chloroquine-sensitive (MRC-pf-20) and -resistant (MRC-pf-303) strains of *P. falciparum *treated with AP and HC, exhibited arrested growth, The IC_50 _values were calculated and found 7.2 μg/ml for AP and 10.8 μg/ml for HC. Maximum growth inhibition (95%) of *P. falciparum *was observed at 62.5 μg/ml and 125 μg/ml concentrations of AP and HC extracts, respectively (Table 1), in both MRC-pf-20 and MRC-pf-303. The activity was further confirmed by a study that showed the parasite stage-specificity of the plant extracts. The herbal extracts were found to arrest the ring stages, which did not revive even after the drugs were withdrawn. These observations confirm that these two plant extracts exert permanent inhibitory activity on the ring stage of the parasite. Further, results of in vivo toxicity study indicated no toxicity associated with the use of these extracts in mice system.

The anti-malarial activity of AP and HC was compared with the recently described herbal drug curcumin. Curcumin shares an almost common range of anti-malarial activity (IC_50_, 6.6 μg/ml) with that of AP (IC_50 _= 7.2 μg/ml) and HC (IC_50 _= 10.8 μg/ml) and was, therefore, taken for combination studies. Isobologram analysis is the most accepted and rigorous method for evaluating drug interactions in combination mixtures and employed in other studies, e.g. cancer [30], tuberculosis [31] inflammation [32] or malaria [7,8,33,34]. Interaction studies for both the plant extracts among themselves (AP+HC) and with curcumin individually (AP+CUR, HC+CUR), at four fixed ratios (1:5, 1:2, 2:1 and 5:1) were carried out in vitro on a chloroquine-resistant isolate of *P. falciparum *(MRC-pf-303). The study revealed the evidence of efficient additive activity between the two indigenous plants (AP+HC). The additiveness, resulting in sum FIC values less than 1 referred to increase of activity when drugs are used in combination. However, curcumin was found to be a potential combination partner with both the indigenous plants (AP and HC) in all four ratios as in both the combinations, the effect was found synergistic. Similar activity was also observed with the test compounds when studied in vivo. Mice treated with the combination of AP and HC showed better survivability compared to the groups that were treated individually with AP or HC.

The search of new compounds from plants is of course an important area of research for exploring new potential drugs for malaria. However, reliable data on the clinical pharmacology, efficacy and safety of such formulae are extremely scarce, preventing a responsible consideration of their potential benefits [35]. Further, lack of awareness among people may worsen the situation particularly when the toxicity aspect of herbal drugs is overlooked. Still there is a need for continued efforts to discover new template molecule from herbal sources.

## Conclusion

This study adds important information to the area of malaria research, which always is in need of alternative anti-malarial drugs and drug combinations to combat with the drug resistant parasites. Although it is premature to conclude at this stage that these herbal combinations can be used as effective anti-malarials, this finding provides a foundation for further exploration of a new effective herbal drug or drug combination with curcumin for protection from the development of resistance among malarial parasites.

## Competing interests

The authors declare that they have no competing interests.

## Authors' contributions

KM and ND have substantial contributions to conception and design of the study. KM has contributed for acquisition and analysis of data. Both ND and KM have participated in drafting the manuscript and revising it. APD and BKS have contributed to critical revision of the manuscript and participated in the coordination of the study. All the authors have read and approved the manuscript.
